# Silibinin inhibits the migration, invasion and epithelial-mesenchymal transition of prostate cancer by activating the autophagic degradation of YAP

**DOI:** 10.7150/jca.63514

**Published:** 2022-09-25

**Authors:** Weichao Dan, Yizeng Fan, Tao Hou, Yi Wei, Bo Liu, Taotao Que, Bixin Yu, Jin Zeng, Lei Li

**Affiliations:** Department of Urology, the First Affiliated Hospital of Xi'an Jiaotong University, Xi'an, Shaanxi 710061, P.R. China.

**Keywords:** silibinin, migration, invasion, epithelial-mesenchymal transition, autophagy, Yes-associated protein

## Abstract

Silibinin (SB), a flavonoid extracted from milk thistle seeds, has been found to exert antitumor effects in numerous tumor types. Our previous study reported that SB had anti-metastatic effects in prostate cancer (PCa). However, the exact underlying molecular mechanisms remain to be determined. The present study aimed to investigate the effects of SB on the migration, invasion and epithelial-mesenchymal transition (EMT) of castration-resistant PCa (CRPC) cells using wound healing, Transwell assays, and western blotting. The results revealed that SB treatment significantly inhibited the migration and invasion of CRPC cell lines. Moreover, SB was confirmed to activate autophagy, as determined using LC3 conversion, LC3 turnover and LC3 puncta assays. Further mechanistic studies indicated that the expression levels of Yes-associated protein (YAP) were downregulated in an autophagy-dependent manner after SB treatment. In addition, the SB-induced autophagic degradation of YAP was associated with the anti-metastatic effects of SB in CRPC. In conclusion, the findings of the present study suggested that SB might inhibit the migration, invasion and EMT of PCa cells by regulating the autophagic degradation of YAP, thus representing a potential novel treatment strategy for metastatic CRPC.

## Introduction

Prostate cancer (PCa) is one of the most common types of malignant tumor in men worldwide, accounting for 21% of all new male adult cancer diagnoses in 2020 1. According to the statistics, there are currently ~1 million new cases of PCa diagnosed worldwide each year 1. In the past few decades, the incidence of PCa in China has rapidly increased from 3.52/100,000 individuals in 1998 to 10.23 /100,000 individuals in 2015, with an average annual growth rate of 11.17% 2. Therefore, PCa poses a serious public health risk among Chinese men. In total, ~1/3 of patients with PCa in China are diagnosed with advanced metastatic PCa, and androgen deprivation therapy is currently an effective treatment option. However, almost all patients will eventually develop into castration-resistant PCa (CRPC) after 12-36 months of treatment 3. Although combination therapy with chemotherapy and novel endocrine treatments are effective in some cases of CRPC, the majority of patients do not respond well; thus, novel effective treatments are urgently required 3. In recent years, scientists have paid particular attention to the effects of traditional Chinese medicine on the treatment of CRPC.

Silibinin (SB), a flavonoid extracted from milk thistle seeds, has been proven in our and other previous studies to exert antitumor effects in a wide variety of cancer types, including bladder 4, renal 5, breast 6, gastric 7, lung 8 and colon cancer 9. Mechanistically, the inhibition of metastasis is considered to be one of the key molecular mechanisms underlying the effects of SB against cancer 10. Our previous study revealed that SB treatment decreased the invasion and migration of renal cell carcinoma both *in vitro* and *in vivo*. In addition, SB attenuated metastasis and epithelial-mesenchymal transition (EMT) in bladder carcinoma 4. Moreover, our research group also discovered that SB inhibited PCa cell invasion and migration by downregulating the expression levels of Vimentin and matrix metalloproteinase-2 (MMP2), which led to the morphological reversal of the EMT phenotype 11. However, the exact underlying mechanisms responsible for the anti-metastatic effects of SB in PCa remain to be determined.

Yes-associated protein (YAP) is the core module of the Hippo signaling pathway and has been found to regulate organ size, stem cell functions, tissue regeneration and tumorigenesis 13. As a transcription factor, YAP translocates to the nucleus to exert its biological functions. Previous studies have reported that YAP was aberrantly upregulated in the majority of patients with PCa, where it served as an oncogene 14,15. Moreover, YAP was found to promote tumor progression and metastasis through its transcriptional enhanced associate domain (TEAD) 16. Therefore, developing targeted biological agents against YAP may represent a promising cancer treatment strategy. Notably, a previous study reported that SB could inhibit the YAP signaling pathway, which subsequently promoted apoptosis in human glioblastoma cells; however, the underlying molecular mechanism was not investigated 17. The present study aimed to investigate the effects of SB on the migration, invasion and EMT of CRPC cells and to determine the underlying molecular mechanisms, with a specific focus on the autophagy-regulated Hippo/YAP signaling pathway. The results revealed that SB could inhibit the migration, invasion and EMT of CRPC cells, which were associated with regulating the autophagic degradation of YAP.

## Materials and methods

### Reagents and antibodies

SB was purchased from Sigma-Aldrich; Merck KGaA and dissolved in DMSO (50 mM). Hydroxychloroquine sulfate (cat. no. H0915) and 3-methyladenine (3-MA; cat. no. M9281) were also purchased from Sigma-Aldrich; Merck KGaA. Rabbit primary antibodies against YAP (cat. no. 14074), Vimentin (cat. no. 5741) and β-actin (cat. no. 4970) were all purchased from Cell Signaling Technology, Inc. Antibodies against LC3B (cat. no. ab48394), E-cadherin (cat. no. ab15148), N-cadherin (cat. no. ab76057) and GAPDH (cat. no. ab181602) were purchased from Abcam.

### Cell lines and culture

The human PCa cell lines, C4-2 and PC-3, were obtained from the American Type Culture Collection and cultured in RPMI-1640 medium (Gibco; Thermo Fisher Scientific, Inc.) supplemented with 10% fetal bovine serum (FBS) (Gibco; Thermo Fisher Scientific, Inc.), 100 U/ml penicillin and 0.1 mg/ml streptomycin (Gibco; Thermo Fisher Scientific, Inc.) at a temperature of 37°C. The intracellular vacuoles of PC-3 and C4-2 cells were captured by an inverted light microscope.

### MTT assay

Briefly, PCa cells were plated into 96-well plates at a density of 5.0×10^4^ cells/ml and treated with different concentrations (0, 25, 50, 75, 100, 125, 150, 175, 200 µM) of SB for 24 or 48 h. DMSO was used as a negative control. Following the incubation, MTT (150 μl) was added/well for a further 4-h incubation at a temperature of 37°C. DMSO was subsequently added/well to dissolve the purple formazan crystals, and cell proliferation was measured using a 96-well microplate reader (Bio-Rad Laboratories, Inc.) at a wavelength of 490 nm. The equation of MTT assay was:

% Cytotoxicity=(100×(Control-Sample))/Control

### Preparation of cytoplasmic and nuclear extracts

Cells at a density of 20.0×10^4^ cells/well were seeded into 6-well plates and treated with SB (50 µM) for 48 h at a temperature of 37°C. DMSO was used as a negative control, the concentration of DMSO was 1‰. The cytoplasmic and nuclear proteins were separated and extracted using a Nuclei EZ Prep Nuclei Isolation kit (Sigma-Aldrich; Merck KGaA) according to the manufacturer's protocol. The expression levels of the different proteins were analyzed using western blotting.

### Western blotting

Cells were treated with SB (0, 25, 50 µM) for 48 h at a temperature of 37°C, then total protein was extracted using RIPA lysis buffer (50 mM Tris, 150 mM NaCl, 0.1% SDS, 1% NP40 and 0.5% sodium deoxycholate; pH 7.4) supplemented with proteinase inhibitors (cat. no. 04693132001; Sigma-Aldrich; Merck KGaA) and phosphatase inhibitors (cat. no. 04906837001; Sigma-Aldrich; Merck KGaA). DMSO was used as a negative control. Total protein was quantified using a Bradford assay and equal amounts (20 µg) of denatured proteins were separated via 10% SDS-PAGE. The separated proteins were subsequently transferred onto polyvinylidene fluoride membranes and blocked with 5% skimmed milk for 1 h at room temperature. The membranes were then incubated with the following primary antibodies overnight at 4°C: Anti-YAP (1:1,000), anti-Vimentin (1:2,000), anti-β-actin (1:5,000), anti-LC3B (1:1,000), anti-E-cadherin (1:2,000), anti-N-cadherin (1:1,000) and anti-GAPDH (1:5,000). Following the primary antibody incubation, the membranes were washed with TBS with 0.1%Tween 20 thrice and incubated with a goat anti-rabbit IgG HRP-conjugated antibody (1:5,000; cat. no. A9169; Sigma-Aldrich; Merck KGaA) for 1 h at a room temperature. Protein bands were visualized using the Clarity Max Western ECL substrate (cat. no. 1705062; Bio-Rad Laboratories, Inc.) and exposure to Bio-Rad's ChemiDoc XRS+ system.

### Immunofluorescence

Cells at a density of 5.0×10^4^ cells/well were plated onto round cover slips and treated with DMSO or SB (50 µM) for 48 h at a temperature of 37°C. Following treatment, the cells were fixed with 4% paraformaldehyde for 15 min at room temperature and permeabilized with 0.5% Triton X- 100 solution. Then cells were blocked with 5% serum for 1 h at room temperature. The cells were subsequently incubated with an anti-YAP primary antibody (1:100) overnight at 4°C. Following the primary antibody incubation, the cells were incubated with a Cy3-conjugated goat anti-rabbit IgG secondary antibody (1:200; cat. no. P0183; Beyotime Institute of Biotechnology) for 1 h at room temperature. Cells were then counterstained with 1 µg/ml DAPI for 5 min at a room temperature. YAP expression was captured with a fluorescent microscope (Olympus, Inc., magnification, ×400).

### Dual-luciferase reporter assay

Cells at a density of 20.0×10^4^ cells/well were seeded into 6-well plates and transfected with monomeric red fluorescent protein (mRFP)-enhanced green fluorescent protein (EGFP)-LC3 reporter plasmids which were mixed with Lipofectamine^®^ 2000 reagent (Invitrogen; Thermo Fisher Scientific, Inc.) and diluted in Opti-MEM (Gibco; Thermo Fisher Scientific, Inc.). After 6 h, medium was changed and maintained for 18 h. Then cells were treated with DMSO or SB (50 µM) for 48 h at a temperature of 37°C. The expression of EGFP and mRFP was detected with a fluorescent microscope (Olympus, Inc., magnification, ×400).

### Wound healing assay

PCa cells were seeded into 6-well plates and cultured with RPMI-1640 medium supplemented with 10% FBS. Upon cells reaching 80-90% confluence, artificial wounds were made by scratching the cell monolayer with a 200‐μl pipette tip. After scratching, the wells were washed twice with PBS to remove the detached cells and cultured with serum‐free RPMI-1640 medium with or without SB (50 µM). DMSO was used as a negative control. The cells were visualized and photographed at 0 and 48 h using an inverted light microscope. The gap distances were semi-quantitatively analyzed using ImageJ 1.48v software (National Institutes of Health).

### Transwell migration and invasion assays

Cells were resuspended in serum-free RPMI-1640 medium and adjusted to a density of 5×10^5^ cells/ml. For the invasion assay, a mixture of RPMI-1640/Matrigel (Sigma-Aldrich; Merck KgaA; duration of precoating, 3 h) containing PCa cells was plated into the upper chamber of Transwell plates. RPMI-1640 medium supplemented with 10% FBS was added to the lower chamber. Following incubation at 37°C for 24 or 48 h, the Transwell plates were washed with PBS, then cells were fixed with 4% paraformaldehyde for 15 min at room temperature and stained with 0.1% aniline violet (dissolved in ethanol) for 15 min at room temperature. Stained cells were visualized using an inverted light microscope (magnification, ×100).

### Small interfering RNA (siRNA/si) and plasmid transfections

siRNAs targeting autophagy related gene 5 (ATG5) and YAP were designed and purchased from Shanghai Gene Pharma Co., Ltd. A negative control (NC) siRNA (si-NC) (Shanghai Gene Pharma Co., Ltd) was also used. The sequences for the siRNAs were as follows: si-ATG5 sequence, 5'-GAAGTTTGTCCTTCTGCTA-3'; si-NC, 5'-UUCUCCGAACGUGUCACGUTT-3'; si-YAP sequence, 5'-GGUGAUACUAUCAACCAAATT-3'. Briefly, 100 pmol siRNAs (si-NC and siATG5) and Lipofectamine^®^ 2000 reagent (Invitrogen; Thermo Fisher Scientific, Inc.) were diluted in Opti-MEM (Gibco; Thermo Fisher Scientific, Inc.) and incubated with the cells at 37°C. Medium was changed after 4-6 hours and then subsequent experiments were conducted. YAP overexpression plasmid (the sequence was inserted into the pcDNA3.1 vector) (Shanghai Gene Pharma Co., Ltd.), and their negative controls (Shanghai Gene Pharma Co., Ltd.) were mixed with Lipofectamine^®^ 2000 (Invitrogen; Thermo Fisher Scientific, Inc.) in Opti-MEM (Gibco; Thermo Fisher Scientific, Inc.). Then the mixture was transfected into cultured PC-3 cells according to standard procedures. The transfection efficiency was analyzed using western blotting following 48 h of transfection.

### Quantitative real-time PCR (qRT-PCR)

After SB treatment (50 µM) for 48 h, total cell RNA was extracted using TRIzol reagent (Invitrogen; Thermo Fisher Scientific, Inc.). DMSO was used as a negative control. Then total RNA was reverse-transcribed using the Primer Script RT reagent kit (Takara Bio, Inc), according to tech manufacturer's protocol. The mRNA levels of YAP, E-cadherin and N-cadherin were detected using qRT-PCR. The thermocycling conditions of the qPT-PCR: an activation stage of 50 °C for 2min and 95 °C for 2min; an amplification stage of 95 °C for 15 s, 55 °C for 30 s, and 72 °C for 30 s for 40 cycles. The sequences of the primers PCR amplification were: 5'-CGAGAGCTACACGTTCACGG-3' (forward) and 5'-GGGTGTCGAGGGAAAAATAGG-3' (reverse) (E-cadherin), 5'-TCAGGCGTCTGTAGAGGCTT-3' (forward) and 5'-ATGCACATCCTTCGATAAGACTG-3' (reverse) (N-cadherin), 5'-TAGCCCTGCGTAGCCAGTTA-3' (forward) and 5'- TCATGCTTAGTCCACTGTCTGT-3' (reverse) (YAP), 5'-GGAGCGAGATCCCTCCAAAAT-3' (forward) and 5'-GGCTGTTGTCATACTTCTCATGG-3' (reverse) (GAPDH). Quantification was calculated using the 2^-∆∆^CT method 18.

### Immunohistochemistry assay

Tumors were embedded with paraffin and sectioned into 5-μm slices. After deparaffinized, the sections were hydrated through a graded ethanol series. Then in methanol supplemented with 3% H_2_O_2_ was used to block endogenous peroxidase activity. The sections were subsequently washed twice with 0.01M PBS and blocked with FBS (Gibco; Thermo Fisher Scientific, Inc.) for 1 h at room temperature. After rinsing, samples were incubated with primary anti-rabbit YAP (1:200, cat. no. 14074, Cell Signaling Technology, Inc.), Vimentin (1:200, cat. no. 5741, Cell Signaling Technology, Inc.) and E-cadherin (1:50, cat. no. ab15148, Abcam) at 4°C overnight, and incubated with the appropriate secondary antibodies (1-3 drops; cat. no. 8114; Cell Signaling Technology, Inc.) at room temperature for 1h. After stained with diaminobenzidine (DAB), the sections were photographed with inverted light microscope (magnification, ×200). Quantitative analysis was performed using ImageJ v1.47 software (National Institute of Health).

### Xenograft animal model

A total of 20 male BALB/c nude mice (weight, 15-20 g; age, 4 weeks) were obtained from the Laboratory Animal Center of Xi'an Jiaotong University (Xi'an, China). All animal experiments were approved by the Biomedical Ethics Committee, Health Science Center of Xi'an Jiaotong University. The health and behavior of the nude mice were monitored daily, high ethical and welfare standards were maintained in all operation involving interactions with animals. The nude mice were housed in a specific pathogen-free environment at a temperature of 22-25°C, with a 12-h light/dark cycle and free access to water and food. PC-3 cells and PC-3 YAP overexpressing cells were resuspended in serum-free RPMI-1640 medium containing Matrigel (Sigma-Aldrich; Merck KGaA) at the density of 2.0×10^7^ cells/ml. In total, 4.0×10^6^ cells in suspension solution were subcutaneously injected into the right flank of nude mice (age, 4 weeks), and upon the tumor volume reaching ~150 mm^3^ in size, the mice were separated into the following four groups: PC-3 control (n=5), PC-3 SB treatment (n=5), PC-3(YAP overexpressing) control (n=5) and PC-3(YAP overexpressing) SB treatment (n=5) groups. When tumor diameter reached 0.3-0.5mm, the mice were received intraperitoneal injection with the DMSO (control groups) and SB 150 mg/kg (SB-treated groups) every 3 days. The tumor volume was calculated using the following formula: Volume (mm^3^) =0.5×length×width^2^. After 30 days, mice were sacrificed by CO_2_ (30% of the chamber volume/min) and tumors were harvested; the animals were exposed to CO_2_ until complete cessation of breathing was observed for 10 min. Tumors were embedded with paraffin for immunohistochemistry staining and western blotting analysis.

### Statistical analysis

Data are presented as the mean ± SD of three independent experiments. Statistical analyses were performed using GraphPad Prism 5.2 software (GraphPad Software, Inc.). Statistical differences between two groups were determined using unpaired Student's t-test and one-way ANOVA. *P*<0.05 was considered to indicate a statistically significant difference.

## Results

### SB inhibits migration, invasion and EMT of CRPC cells

**Figure 1:**
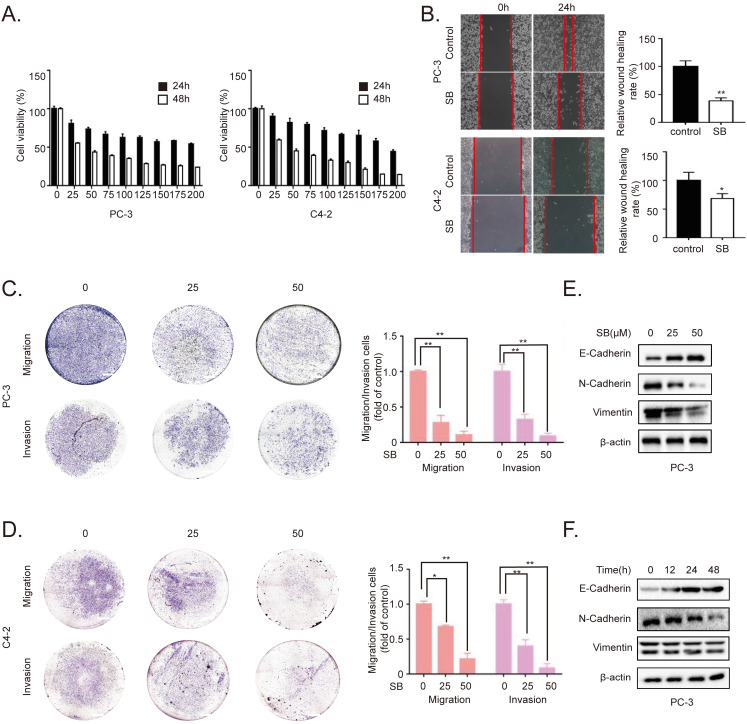
SB inhibits the migration, invasion and EMT of prostate cancer cells. (A) PC-3 and C4-2 cells were treated with different concentrations of SB for 24 or 48 h, and the cell viability was analyzed using a MTT assay. (B) Wound healing assay was performed using PC-3 and C4-2 cells following the treatment with 50 µM SB for 48 h. (C) PC-3 and C4-2 cells were treated with 0, 25 or 50 µM SB and Transwell (C) migration and (D) invasion assays were performed. Magnification, x100; scale bar, 200-µm. ^**^P<0.01. (E) PC-3 cells were treated with 0, 25 or 50 µM SB for 48 h. The protein expression levels of E-cadherin, N-cadherin and Vimentin (typically running as a doublet) were analyzed using western blotting. (F) PC-3 cells were treated with 50 µM SB for 0, 12, 24 or 48 h. The protein expression levels of E-cadherin, N-cadherin and Vimentin (typically running as a doublet) were analyzed using western blotting. SB: silibinin.

To determine the inhibitory effects of SB, CRPC cell lines PC-3 and C4-2 were treated with different concentrations of SB. As shown in Fig. 1A, SB treatment inhibited the proliferation of CRPC cells in a concentration- and time-dependent manner, with an IC_50_ value of 30 µM in PC-3 cells and 42 µM in C4-2 cells at 48 h. Furthermore, we detected the effects of SB on cell viability of human benign prostatic hyperplasia cell line BPH-1. The results showed that silibinin didn't have significant inhibitory effects on cell viability of BPH-1. (Fig. S1). Wound healing and Transwell assays were subsequently performed to determine the effect of SB on the migratory and invasive abilities of CRPC cells. The results revealed that SB inhibited the migration and invasion of CRPC cells in a concentration-dependent manner (Fig. 1B-D). It is well known that EMT plays important roles in cancer metastasis 19. To investigate the effect of SB on the EMT of CRPC, western blotting was used to analyze the expression levels of EMT-related markers, E-cadherin, N-cadherin and Vimentin, in PC-3 cells. The results demonstrated that the expression level of the epithelial marker, E-cadherin, was gradually upregulated, while the expression levels of the mesenchymal markers, N-cadherin and Vimentin, were gradually downregulated following the treatment with SB in both a dose- and time-dependent manner (Fig. 1E and F). To further determine if SB could affect EMT through transcriptional regulation, we performed qRT-PCR to detect message RNA (mRNA) level of EMT-related markers (Fig. S2). The results showed that SB significantly upregulated E-cadherin transcript levels and downregulated N-cadherin transcript levels. These results indicate that SB may inhibit the migration, invasion and EMT of CRPC cells.

### SB induces autophagy in CRPC cells

**Figure 2:**
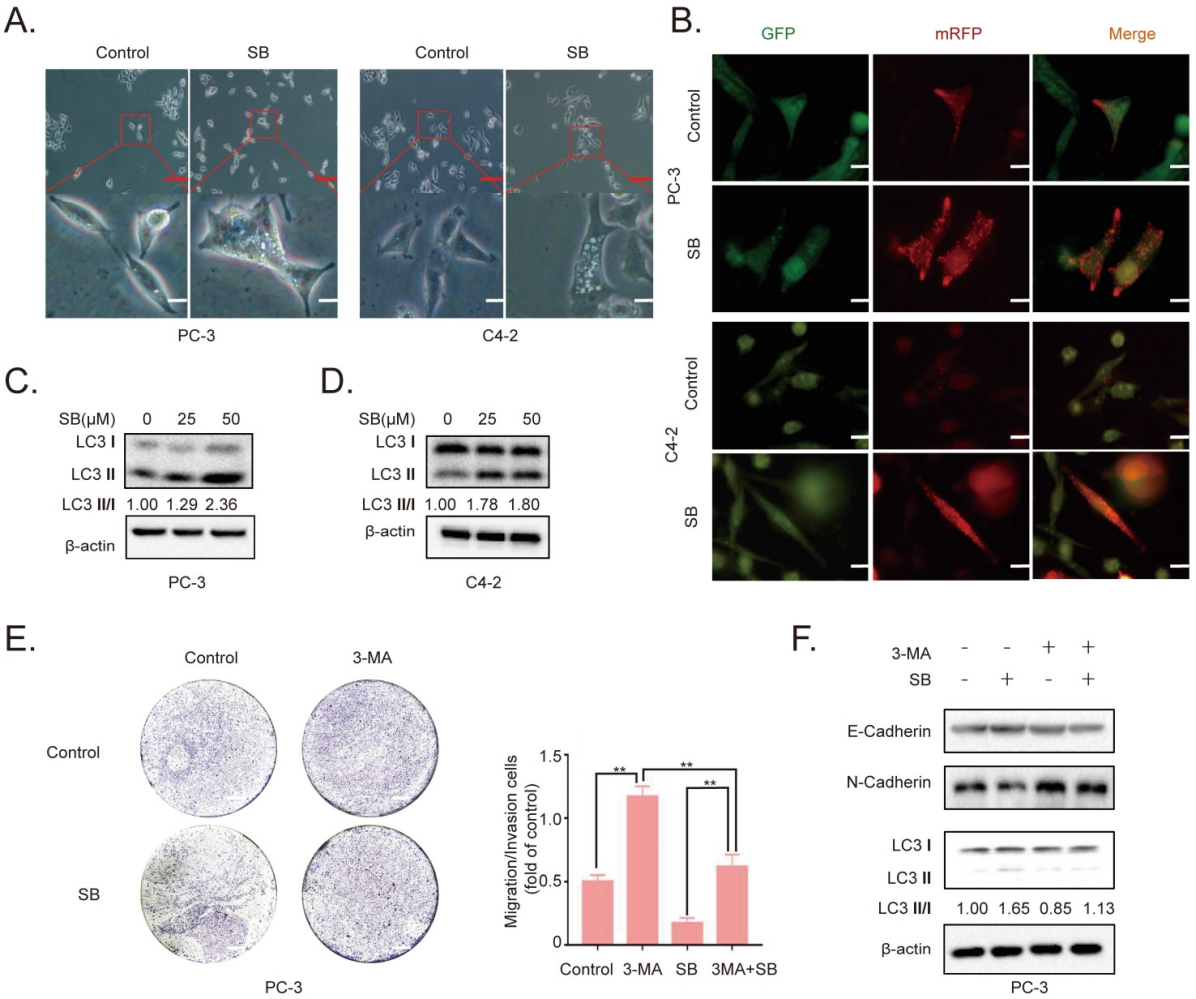
Effect of SB on autophagy in prostate cancer cells. (A) PC-3 and C4-2 cells were treated with 50 µM SB for 48 h and then observed under an inverted light microscope. The red box corresponds to the image with a higher magnification ratio. Upper scale bar, 200-µm; lower scale bar, 50-µm. (B) PC-3 and C4-2 cells were transfected with mRFP-EGFP-LC3 reporter plasmids and treated with 50 µM SB for 48 h. Visualization of LC3 puncta was performed using a fluorescence microscope. Scale bar, 50-µm. (C) PC-3 and (D) C4-2 cells were treated with 0, 25 or 50 µM SB for 48 h. The protein expression levels of LC3 were analyzed using western blotting. (E) Transwell migration assays were performed in PC-3 cells following the treatment with 50 µM SB and/or 5 mM 3-MA. Magnification, x100; scale bar, 200-µm. ^**^P<0.01. (F) PC-3 cells were treated with 50 µM SB and/or 5 mM 3-MA. The protein expression levels of E-cadherin, N-cadherin and LC3 were analyzed using western blotting. SB: silibinin; mRFP-EGFP-LC3: monomeric red fluorescent protein-enhanced green fluorescent protein-LC3.

To determine the antitumor mechanism of SB in CRPC, PC-3 and C4-2 cells were incubated with 50 µM SB for 48 h and morphological changes were subsequently visualized. The number of intracellular vacuoles was significantly increased in PC-3 and C4-2 cells following SB treatment compared with the control group (Fig. 2A). To further determine the association between the formation of intracellular vacuoles and cell autophagy, monomeric red fluorescent protein (mRFP)-enhanced green fluorescent protein (EGFP)-LC3 reporter plasmids were transfected into PC-3 and C4-2 cells. As shown in Fig. 2B, the number of yellow LC3 and red LC3 puncta was increased in CRPC cells treated with SB, indicating that SB could activate autophagy. The expression levels of LC3 were further analyzed using western blotting, and the results showed that LC3-II expression levels were upregulated following SB treatment (Fig. 2C and D). In addition, inhibition of autophagy by 3-MA (which had little impact on CRPC cells proliferation, shown in Fig. S3) significantly attenuated SB-induced inhibition of migration (Fig. 2E) and EMT (Fig. 2F), suggesting an important role for autophagy in the anticancer effects of SB. These results indicate that SB may activate autophagy, and the inhibition of autophagy may attenuate SB-induced antitumor effects in CRPC cells.

### SB promotes the autophagic degradation of YAP

**Figure 3:**
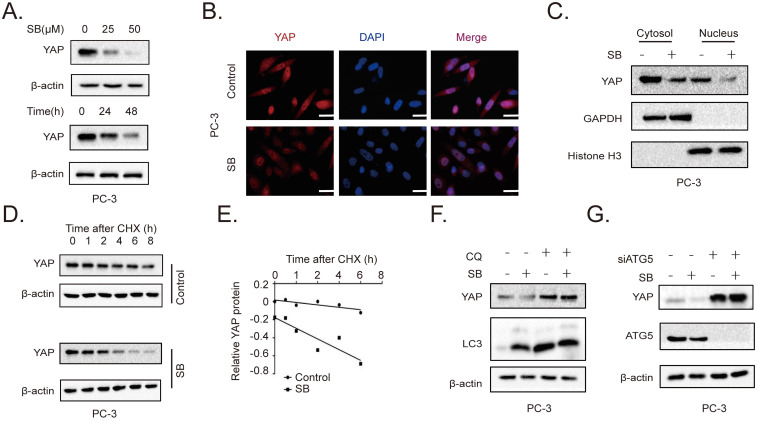
SB promotes the autophagic degradation of YAP. (A) PC-3 cells were treated with 0, 25 or 50 µM SB for 48 h or with 50 µM SB for 0, 24 or 48 h. The protein expression levels of YAP were analyzed using western blotting. (B) PC-3 cells were treated with 50 µM SB for 48 h. Immunofluorescence was used to analyze the expression of YAP in PC-3 cells. Scale bar, 50-µm. (C) PC-3 cells were treated with 50 µM SB for 48 h. The cytosolic and nuclear protein expression levels of YAP were detected using western blotting. (D) PC-3 cells were treated with 50 µM SB and/or 100 µg/ml CHX. The protein expression levels of YAP were analyzed using western blotting. (E) Semi-quantification of the band intensities from part (D). YAP protein bands were normalized to β-actin, then normalized to the 0 h time point. (F) PC-3 cells were treated with 50 µM SB and/or 50 µM CQ. The protein expression levels of YAP and LC3 were analyzed using western blotting. (G) PC-3 cells were treated with 50 µM SB and/or transfected with si-ATG5. The protein expression levels of YAP and ATG5 were analyzed using western blotting. SB: silibinin; YAP: Yes-associated protein; CHX: cycloheximide; CQ, chloroquine; ATG5: autophagy related gene 5; si: small interfering RNA.

To further determine the role of the YAP signaling pathway in the SB-mediated antitumor effects on CRPC cells, the expression of YAP was determined. The results revealed that SB treatment downregulated YAP expression levels in a concentration- and time-dependent manner in PC-3 cells (Fig. 3A). Similar results were obtained from the immunofluorescence assay (Fig. 3B, Fig. S4). A previous study reported that YAP exerted its functions by entering the nucleus 20. As shown in Fig. 3C, SB treatment decreased both the cytoplasmic and nuclear expression of YAP. The decrease YAP level could be caused by either decreased synthesis or enhanced degradation by the tumor cells. By examining the YAP transcript level in PC-3 cells cultured with or without SB, we found that SB increased YAP mRNA expression, suggesting that SB might promote YAP protein degradation (Fig. S5). To further explore the mechanism of SB-induced downregulation of YAP, cycloheximide (CHX) was used to inhibit the translation of proteins. PC-3 cells were treated with 50 µM SB for 40 h, and then incubated with 100 µg/ml CHX for 0, 1, 2, 4, 6, and 8 h. The results revealed that YAP expression levels were downregulated to a greater extent in combined treatment group, comparing with CHX treatment alone (Fig. 3D and E). To determine the autophagic mechanism of YAP downregulation, chloroquine (CQ) and si-ATG5 were used to inhibit autophagy-dependent protein degradation. As shown in Fig. 3F and G, inhibition of autophagy attenuated the SB-mediated downregulation of YAP expression. These results indicate that SB may induce the autophagic degradation of YAP in CRPC cells.

### SB inhibits the migration, invasion and EMT of CRPC cells by downregulating YAP expression

**Figure 4:**
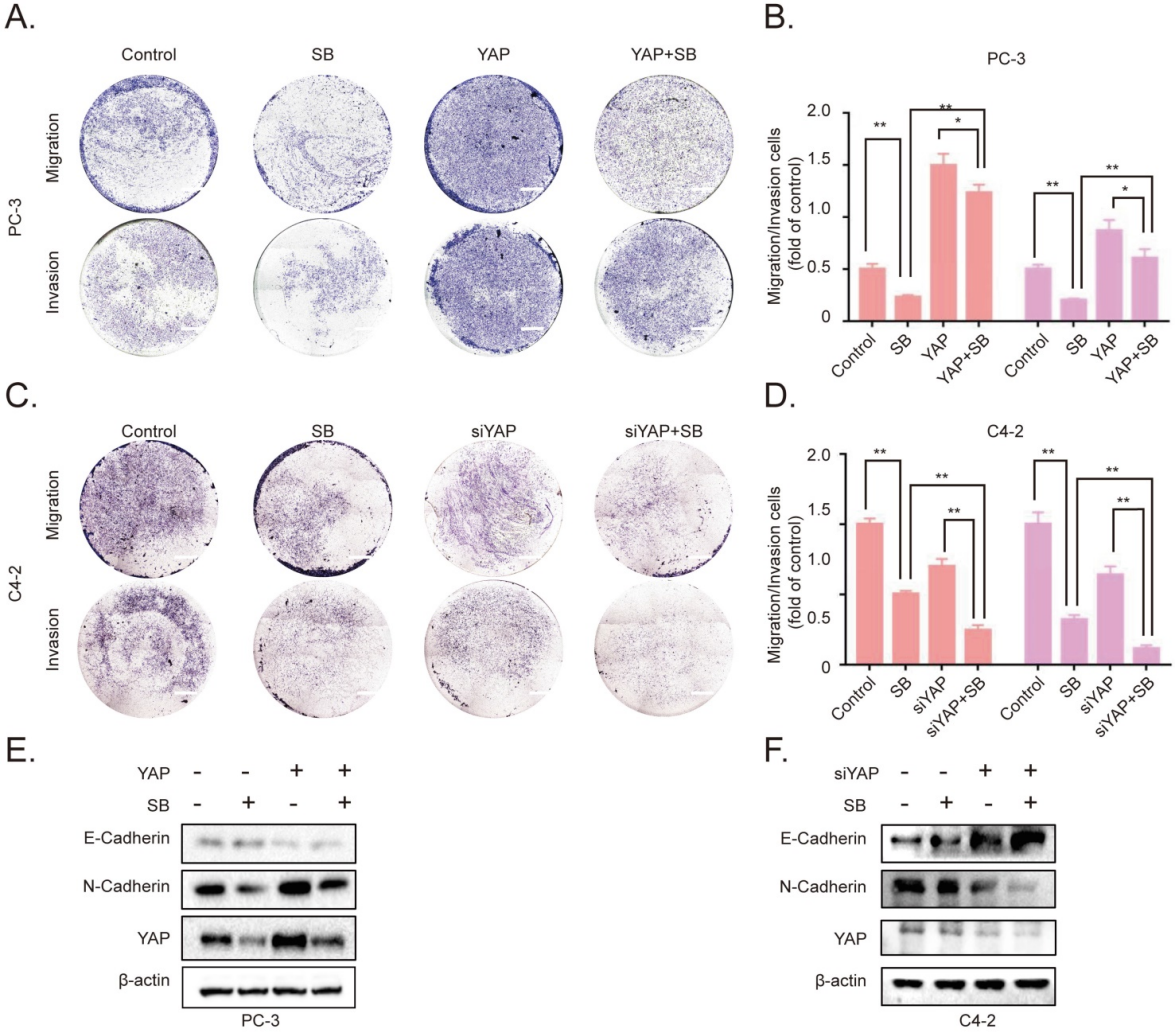
SB inhibits the migration, invasion and epithelial-mesenchymal transition of prostate cancer cells by downregulating YAP expression. Transwell (A, B) migration and invasion assays were performed with YAP-overexpressing PC-3 cells. Cells were treated with 50 µM SB for 48 h. Magnification, x100; scale bar, 200-µm. ^*^P<0.05, ^**^P<0.01. Transwell (C, D) migration and invasion assays were performed with YAP-knockdown C4-2 cells. Cells were treated with 50 µM SB for 48 h. Magnification, x100; scale bar, 200-µm. ^**^P<0.01. (E) YAP-overexpressing PC-3 cells were treated with 50 µM SB for 48 h. The protein expression levels of E-cadherin, N-cadherin and YAP were analyzed using western blotting. (F) C4-2 cells were treated with siYAP and SB (50 µM) for 48 h. The protein level of E-cadherin, N-cadherin and YAP were detected by western blotting. SB: silibinin; YAP: Yes-associated protein; si, small interfering RNA.

To further determine the association between SB-induced YAP degradation and the migration, invasion of CRPC cells, we established YAP-overexpressing PC-3 cells. The results of the Transwell assay demonstrated that the overexpression of YAP attenuated the effects of SB (Fig. 4A and B). Next, siRNA was used to silence YAP expression in C4-2 cells. As expected, the knockdown of YAP further enhanced the inhibitory effects of SB treatment (Fig. 4C and D). The underlying inhibitory effects of SB on EMT and YAP were subsequently explored. As shown in Fig. 4E and F, the results found that YAP overexpression attenuated the suppressive effects of SB, while YAP knockdown promoted the suppressive effects of SB.

### Antitumor effects of SB on CRPC cells in vivo

**Figure 5:**
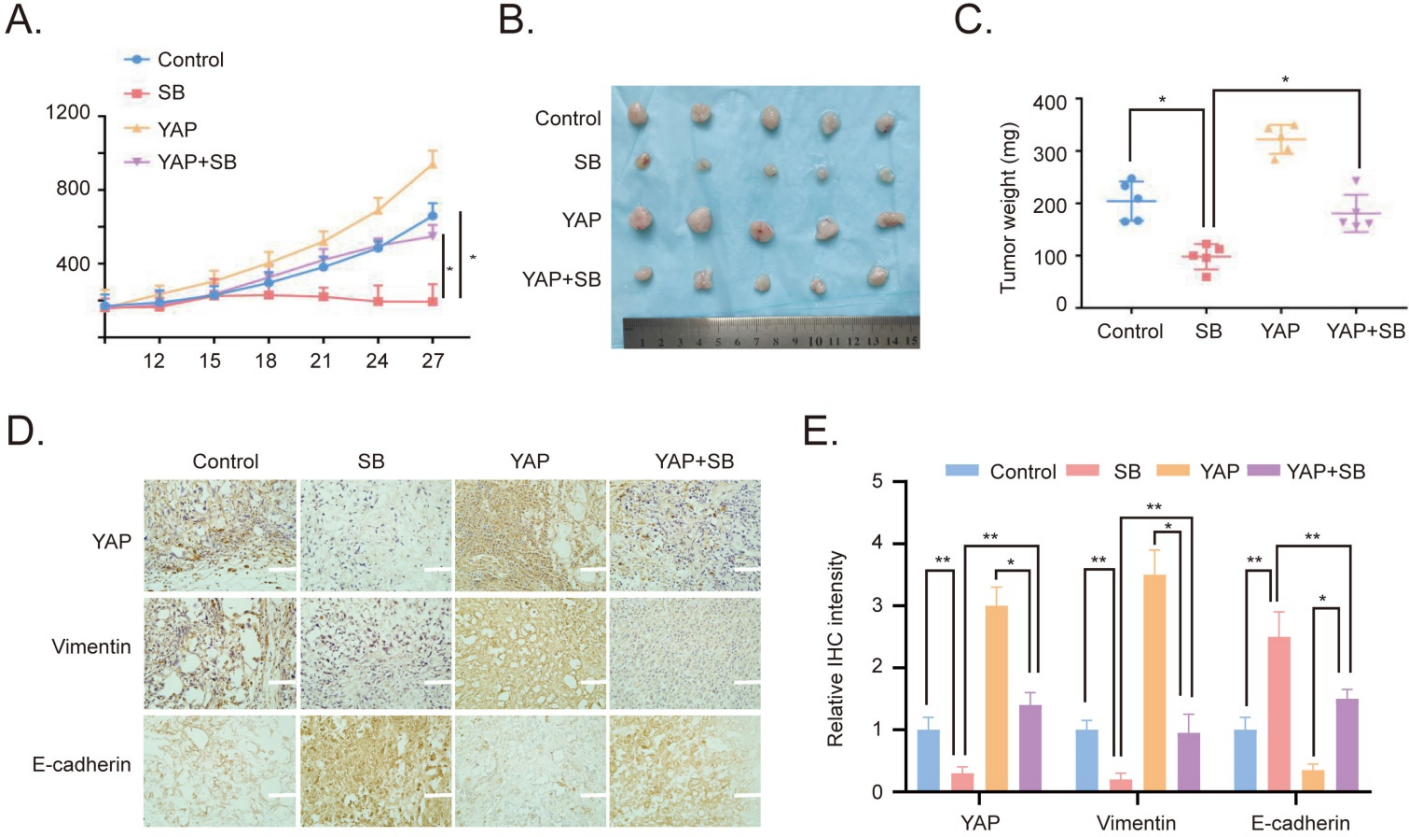
Antitumor effects of SB on prostate cancer cells *in vivo*. (A) Tumor volumes from mice were measured every 3 days. (B) Images of the subcutaneous xenograft tumors formed from the different groups following treatment with SB for 30 days. (C) Weight of the dissected xenografts tumors. n=5 mice/experimental group. The data are presented as the mean ± SD. ^*^P<0.05. (D) Expression levels of E-cadherin, Vimentin and YAP in the tumors were analyzed using immunohistochemistry. Scale bar, 100-µm. (E) Quantification of the protein expression from part (D). SB: silibinin; YAP: Yes-associated protein.

To verify the effects of SB *in vitro*, YAP-overexpressing PC-3 cells were used to establish a xenograft tumor model in male BALB/c nude mice. The results revealed that the overexpression of YAP increased the growth of the xenograft tumors, while SB treatment had a negative effect on tumor growth (Fig. 5A). Similarly, the weight of the tumors in the YAP-overexpressing group was the largest, while the tumor weight in the SB treatment group was decreased compared with the other groups (Fig. 5B and C). In addition, the expression levels of E-cadherin, Vimentin and YAP in the tumors were analyzed using immunohistochemistry staining. The results demonstrated that the expression levels of YAP and Vimentin were downregulated following SB treatment, while SB treatment upregulated E-cadherin expression levels (Fig. 5D and E). These results suggested that, at least to some extent, SB may be able to reverse the promoting effects of YAP on EMT *in vivo*.

## Discussion

In the past few decades, scientists have been focused on exploring effective strategies to treat human cancers and searching for novel antitumor drugs^21^. A large number of drugs have been proven to exert a wide range of antitumor effects and are currently used in the clinic, including plant-derived drugs, targeted therapies and chemotherapy drugs. Among them, SB, a flavonoid compound extracted from *Silybum marianum*, has been suggested as a promising novel treatment for numerous cancer types 22-24. SB has been reported to exert antitumor effects in breast carcinoma 25, hepatocellular carcinoma 26, bladder cancer 27, PCa 28 and renal cell carcinoma 29.

CRPC, the main reason for PCa metastasis, is inevitable to occur for most PCa patients after initial androgen-deprivation therapy. Different from untreated (hormone-naive) prostate adenocarcinoma, CRPC is characterized by a peculiar genetics molecular landscape. CRPC patients exhibit the main characteristics of hormone-independent, phenotypic plasticity, a degree of stemness signatures, chemotherapy resistance and high rates of metastasis 30. In the past several years, many novel agents have been designed and applied to treat CRPC, but a considerable number of patients still face the problem of primary resistance to new generation agents 31. As novel natural compounds, SB has been confirmed to have potential antitumor effects on multiple cancers, including PCa 28. Despite increasing evidence demonstrating a solid molecular and mechanistic level of positive activity of SB on prostate cancer, its precise effects and underlying mechanism in CRPC are not completely elucidated. The results of the present study revealed that SB inhibited the migration and invasion of CRPC *in vitro.* Furthermore, the present data demonstrated that SB treatment upregulated E-cadherin expression levels, which is an epithelial marker 32, and downregulated N-cadherin and Vimentin expression levels, which are mesenchymal markers 32. These results indicated the important inhibitory role of SB in CRPC.

Autophagy is a cellular process that involves transporting cellular materials to lysosomes for degradation 33. As a self-digestion mechanism, autophagy has been discovered to play important roles in numerous diseases types, particularly in cancer 34. Previous studies have reported that autophagy suppressed tumorigenesis via multiple possible mechanisms 35,36. In prostate and breast cancer, the expression of several essential autophagy genes was found to be partially downregulated, such as Beclin-1 (BECN1) 37,38. Further studies have also demonstrated that the suppression of autophagy promoted cancer cell proliferation 39-41. Previous studies showed that BECN1 heterozygous mutant mice were more susceptible to tumor development 42-44. Moreover, accumulating evidence verified that autophagy could inhibit EMT. EMT regulators Snail and Slug were upregulated in glioblastoma cells after autophagy inhibition by silencing Beclin-1, leading to enhancement of migration and invasion 45. Vice versa, nutrient deprivation-mediated autophagy induction could suppress the mesenchymal phenotype and tumor metastasis 46. Additionally, it was reported that death effector domain-containing DNA-binding protein (DEDD) physically interacted with the class III PtdIns 3-kinase complex, which controlled the initiation of autophagy. This interaction activated autophagy and induced the autophagy-mediated lysosomal degradation of Snail and Twist, two master inducers of the EMT process 47,48. Therefore, autophagy is generally considered as a tumor-suppressive mechanism and targeting autophagy has been hypothesized to represent a promising cancer treatment strategy. The findings of the present study revealed that SB treatment induced autophagy in PCa cells, which was verified using inverted microscopy, a LC3 turnover assay and western blotting. In addition, SB-mediated inhibition of migration and invasion was found to be closely associated with autophagy. Pretreatment with 3-MA, an inhibitor of autophagy, partially rescued SB-induced anti-metastatic effects in PCa cells.

As a transcriptional co-activator that mediates multiple biological functions, YAP has been discovered to be essential for cancer initiation, progression and metastasis in the majority of tumor types 49. In PCa, previous studies have identified that YAP was hyperactivated and drove tumorigenesis and development 30,50,51. Another study found that the expression levels of the YAP inhibitors, LATS1/2, were significantly downregulated in metastatic PCa compared with non-metastatic PCa, which indicated a role for YAP in PCa metastasis 52. Several treatments targeting YAP signaling pathways have shown therapeutic potential in CRPC and other types of urological cancer 53. Verteporfin, an inhibitor of the YAP/TEAD interaction, was found to inhibit the proliferation of CRPC 54. Sphingomab, a sphingosine-1-phosphate antagonist that inhibited YAP transcriptional activity, was discovered to attenuate the metastasis of bladder cancer cells 55. In the present study, SB treatment downregulated the protein expression levels of YAP, which resulted in the inhibition of the invasion, migration and EMT of CRPC cells. Moreover, the current data found that post-translational regulation was the main underlying mechanism of the SB-induced downregulation of YAP expression. Previous studies have reported that YAP was degraded through the autophagic-lysosomal 56-58 and ubiquitin system 59-61. The present study used CQ and si-ATG5 to inhibit protein degradation via the autophagy-lysosomal pathway. The expression levels of YAP were significantly upregulated when autophagy was blocked by CQ or si-ATG5. Furthermore, blocking autophagy also attenuated the inhibitory effects of SB on the migration, invasion and EMT of CRPC. Therefore, the present research discovered a potential novel mechanism of autophagy-dependent degradation of YAP that was mediated by SB in CRPC. Basically, SB is reported to modulate invasion, migration and EMT phenotype in cancers through multiple mechanisms, such as AR signaling 62 and Wnt/β-catenin pathway 63. Our present study proposed a novel viewpoint which partially contributed to SB-mediated cancer inhibition. However, whether the SB-mediated downregulation of YAP expression is also dependent on other mechanisms, and the underlying association between YAP and metastatic features requires further investigations.

In conclusion, the findings of the present study suggested that SB might promote the degradation of YAP by activating autophagy, which highlighted a potential novel mechanism for the antitumor effects of SB in CRPC. These findings indicate that SB may represent an effective agent for CRPC treatment.

## Supplementary Material

Supplementary figures.
