# Supplementary material for: Silibinin inhibits the migration, invasion and epithelial-mesenchymal transition of prostate cancer by activating the autophagic degradation of YAP
Source: J Cancer. 2022 Sep 25;13(13):3415–26. doi: 10.7150/jca.63514 (PMC9608209; doi:10.7150/jca.63514)
Supplement: Supplementary file 1 — Supplementary figures. [file jcav13p3415s1.pdf]

Supplemental Figures

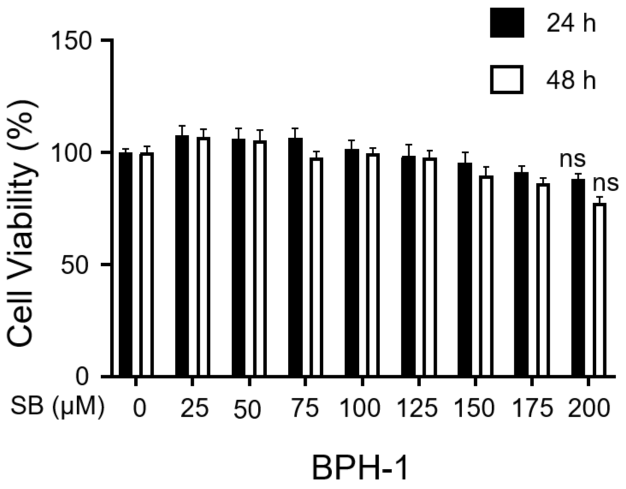

Fig. S1 Cell viability of SB on benign prostatic hyperplasia cell line BPH-1.

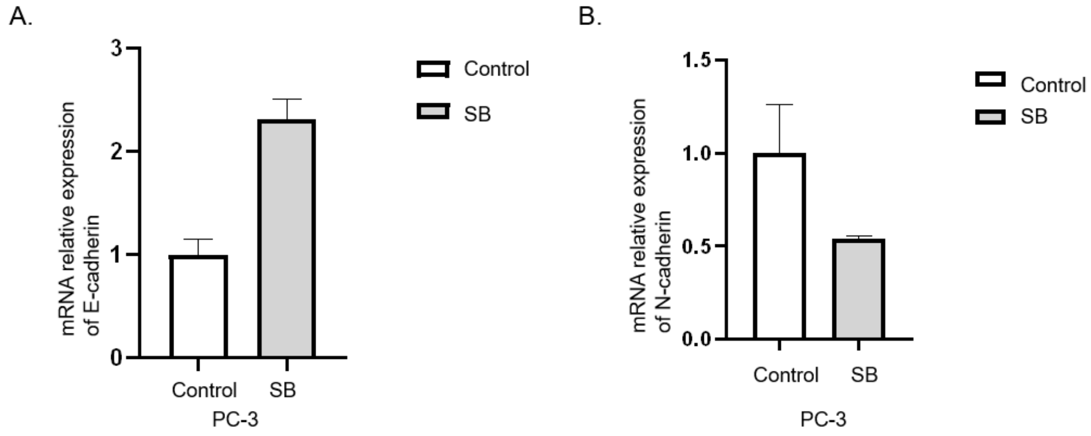

Fig. S2 A, B, mRNA relative expression of E-cadherin and N-cadherin detected with qRT-PCR. Cells were treated with SB (50 μM) for 48 h. SB: silibinin.

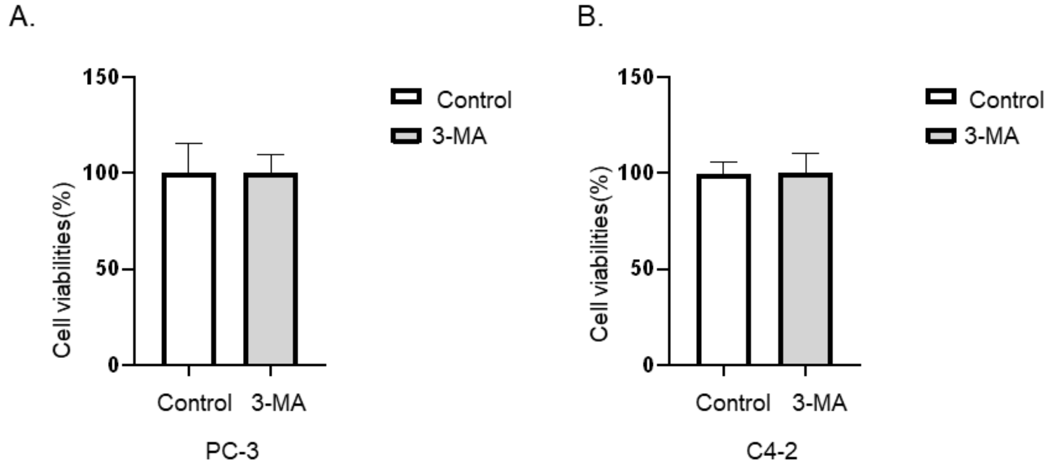

Fig. S3 A, B, Cell viabilities of PC-3 and C4-2 were detected with MTT assay. Cells were treated with 3-MA (50mM) for 24h. 3-MA: 3-Methyladenine.

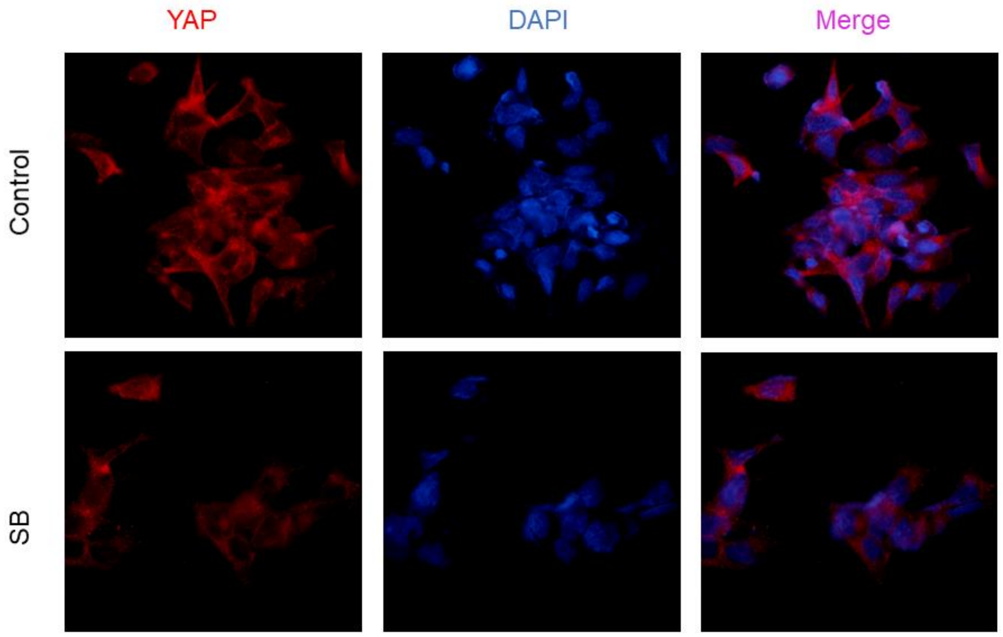

Fig. S4 C4-2 cells were treated with SB (50 μM) for 48 h. Immunofluorescence was used to detect the expression of YAP in C4-2 cells. SB, silibinin; YAP: Yes-associated protein.

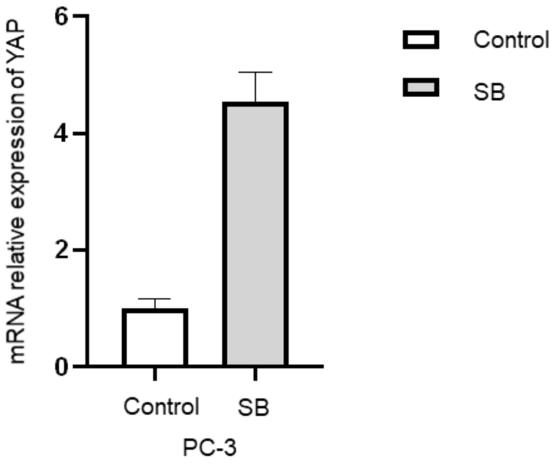

Fig. S5 mRNA relative expression of YAP detected with qPCR. Cells were treated with SB (50 μM) for 48 h. SB, silibinin; YAP: Yes-associated protein.
